# Adjuvant effects of Chinese medicinal tonics on gastric, liver, and colorectal cancers—OMICs-based contributions to understanding their mechanism of action

**DOI:** 10.3389/fphar.2022.986765

**Published:** 2022-11-29

**Authors:** Zhigang Zuo, Jia Jia, Hongliang Li, Run Shi, Di Wang, Ke-Wu Zeng, Hong Nie, Xin-Guo Wang, Wen Liu, Minglun Li, Yibin Feng, Xuan Bin Wang

**Affiliations:** ^1^ Laboratory of Chinese Herbal Pharmacology, Department of Pharmacy, Renmin Hospital, Biomedical Research Institute, School of Pharmaceutical Sciences and Hubei Key Laboratory of Wudang Local Chinese Medicine Research, Hubei University of Medicine, Shiyan, China; ^2^ Department of Oncology, The First Affiliated Hospital of Nanjing Medical University, Nanjing, China; ^3^ Engineering Research Center of Chinese Ministry of Education for Edible and Medicinal Fungi, Jilin Agricultural University, Changchun, China; ^4^ State Key Laboratory of Natural and Biomimetic Drugs, School of Pharmaceutical Sciences, Peking University, Beijing, China; ^5^ International Cooperative Laboratory of Traditional Chinese Medicine Modernization and Innovative Drug Development of Chinese Ministry of Education (MOE), Guangdong Province Key Laboratory of Pharmacodynamic Constituents of TCM and New Drugs Research, College of Pharmacy, Jinan University, Guangzhou, China; ^6^ School of Pharmacy, Hebei University of Chinese Medicine, Shijiazhuang, China; ^7^ School of Pharmacy, Guizhou Medical University, Guiyang, China; ^8^ Department of Radiation Oncology, University Hospital, LMU Munich, Munich, Germany; ^9^ School of Chinese Medicine, The University of Hong Kong, Pokfulam, Hong Kong SAR, China

**Keywords:** Chinese medicines, tonics, gastrointestinal cancer, OMICs, hallmark

## Abstract

Gastric, liver, and colorectal cancers belong to gastrointestinal (GI) cancers, one of the most threatening diseases in the world. The tonics class in Chinese medicines plays a critical role in antigastrointestinal cancer as adjuvants. However, it is a challenge to study the effects and underlying mechanisms of tonics due to their multiple components and multiple targets; OMICs were introduced to facilitate the investigation of the complex mixture of tonics. In this review, the online databases PubMed, ProQuest, Web of Knowledge, China National Knowledge Infrastructure (CNKI), Chongqing VIP, and Wanfang were retrieved from 1 January 2011 to 31 May 2022, in an aim to summarize and discuss the research progress of the effects and, especially, the underlying mechanisms of tonics for antigastrointestinal cancers *via* OMICs. The results showed that through the combination of OMICs and other technologies, tonics have been used for gastrointestinal cancer by targeting cancer hallmarks, enhancing body resistance to carcinogenesis, enhancing therapeutic effects, and/or decreasing side effects. In conclusion, tonics may play a promising role in gastric, liver, and colorectal cancers as adjuvants and can be well investigated *via* the combination of OMICs and other technologies, which deserves further study.

## 1 Introduction

Gastrointestinal (GI) cancers are one of the most threatening diseases in the world. There were approximately 5,142,192 new cases and 3,628,920 deaths from GI cancers in the world in 2020 (Sung et al., 2021). Based on disease sites, GI cancers are divided into two families: upper digestive tract cancers (including esophageal, stomach, pancreatic, liver, gallbladder, and lymphoma involving the mucosa-associated lymphoid tissue, gastrointestinal stromal, and biliary tree) and lower cancers (including colorectal, anal, and gastrointestinal carcinoid). The order of the mortality rate from high to low for cancer sites was liver (8.3%), stomach (7.7%), colon (5.8%), esophagus (5.5%), pancreas (4.7%), rectum (3.4%), gallbladder (0.9%), and anus (0.2%) (Sung et al., 2021). The total new deaths of GI cancers (36.7%) exceeded those of lung cancer (18.0%) and GI cancers ranked as the leading cause of death (Sung et al., 2021). The conventional treatments of GI cancers include surgical resection, chemotherapy, radiotherapy, targeted therapy, and immunotherapy. However, some patients cannot tolerate surgical resection. Chemotherapy and radiotherapy induce toxicity and side effects. Targeted therapy and immunotherapy are often expensive, especially for patients in developing countries. Thus, natural medicines have drawn attention due to their lower toxicity and effectiveness as adjuvant strategies and have been widely used in clinical practice to adjust patients’ constitution and reduce toxicity and side effects after surgery, chemotherapy, and radiotherapy.

**FIGURE 1:**
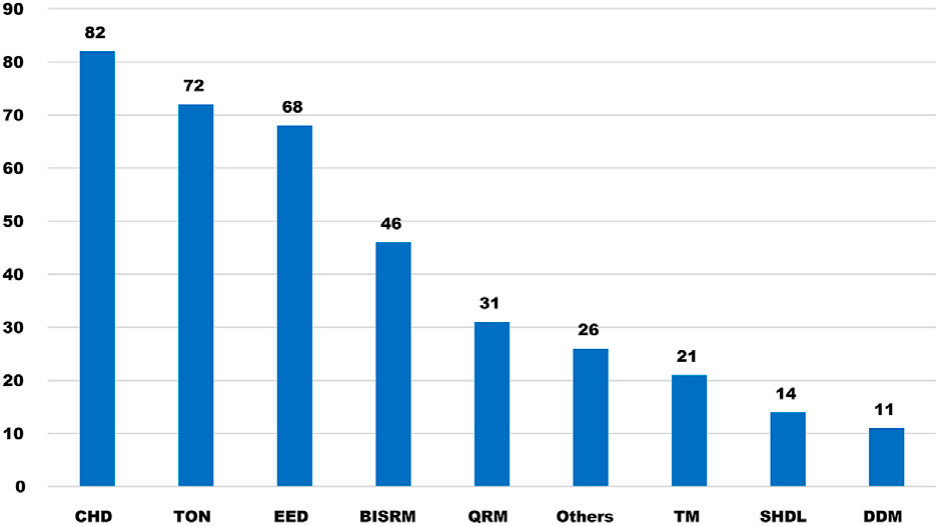
Category of anticancer CMs. CHD, medicines that clear heat and detoxify; TON, tonics; EED, expectorants and damp-expelling medicines; BISRM, blood invigorating and stasis resolving medicines; QRM, Qi-regulating medicines; TM, toxic medicines; SHDL, soften hardness and dissolve lump; and DDM, damping–draining medicines.

Tonics of Chinese medicines (CMs) refer to medicines that can supplement Qi, blood, Yin, and Yang of the human body, relieve deficiency and weakness syndromes, enhance visceral function, and improve the body’s ability to resist disease. Their pharmacological actions include enhancing immunofunction, regulating metabolism of substances, improving the endocrine system, anti-aging, and anticancer (Chen, 2017). Tonics are divided into four categories, Qi tonics (e.g., *Panax ginseng* C.A.Mey and *Astragalus membranaceus* (Fisch.) Bge. var. mongholicus (Bge.) Hsiao), blood tonics (e.g., *Angelica sinensis* (Oliv.) Diels and *Polygonum multiflorum* Thunb.), Yin tonics (e.g., *Lycium barbarum* L. and *Ophiopogon japonicus* (L. f) Ker-Gawl.), and Yang tonics (e.g., *Epimedium brevicomu* Maxim.) (Chen, 2017). Some Chinese formulas are also believed to be tonics such as Liu Wei Di Huang pills (Xiong et al., 2022). There are 72 tonics out of 371 CMs for anti-GI cancers, which ranked the second class of the most frequently used CMs following the clearing heat and detoxifying class (Xu et al., 2018) (Figure 1).

However, it was difficult for the research community to explore the effects, and particularly the underlying mechanisms of tonics due to their multiple components (Yang et al., 2018; Yang et al., 2019), complex pharmacokinetic (PK) processes (Wang et al., 2021a; Wang et al., 2021b), and multiple targets until OMICs were introduced (Li et al., 2019; Wang et al., 2019; Wang and Lu, 2019; Li et al., 2020; Liu et al., 2021a; Li et al., 2022b). OMICs are novel technologies that have been dramatically developed in the last 2 decades. Small size samples and large-scale and high-throughput screening make OMICs possible to apply to various disciplines in biology including complex pharmacological mechanisms (Li et al., 2022a). OMICs include genomics, proteomics, metabolomics (or metabonomics), metagenomics, transcriptomics, epigenomics, glycomics, and lipomics. In this review, we retrieved online databases, aiming to summarize and discuss the OMICs-based research progress of adjuvant effects, especially underlying mechanisms, and provide insights into the complex multiple components and targets of tonics on GI cancers.

## 2 Materials and methods

### 2.1 Data retrieval and collection

The keywords “OMICs or genomics/proteomics/metabolomics/metabonomics/metagenomics/transcriptomics/ epigenomics/glycomics/lipomics” and “tonics” and “gastrointestinal cancer” were used to retrieve studies of tonics for GI cancers from the online databases of PubMed, ProQuest, Web of Knowledge, China National Knowledge Infrastructure (CNKI), Chongqing VIP, and Wanfang from 1 January 2011 to 31 May 2022. Duplicates were discarded. The effects, overall efficacy, and underlying mechanisms *via* OMICs technologies in these studies were summarized and analyzed. All plant names were checked with the World Flora Online (www.worldfloraonline.org) or MPNS (http://mpns.kew.org).

### 2.2 Inclusion criteria

The inclusion criteria were as follows (Ding et al., 2019):

• The contents of the literature involve the *in vitro*, *in vivo*, and clinical effects of tonics on GI cancers
• The references included pure compounds, single herbal fractions, and formulas of tonics
• The methodologies were designed using OMICs

### 2.3 Exclusion criteria

The exclusion criteria were as follows (Ding et al., 2019):

• The literature was associated with neither tonics nor OMICs
• The pure compounds were not naturally from tonics but were chemical derivatives
• The species of tonics were not clearly presented, or the plant names were not checked in the “World Flora Online” (www.worldfloraonline.org) or MPNS (http://mpns.kew.org)
• The fractions and/or formulas of tonics were described with neither the extraction methodology nor quality control
• The components of the formulas were not given
• The concentration/dose of tonics was not given
• The clinical studies were not randomized and controlled
• The in vivo and clinical studies did not claim any ethical approvals, and the clinical studies were conducted without the declaiming of patients’ agreement or signing informed consent

## 3 OMICS for adjuvant effects and mechanisms of tonics on gastric, liver, and colorectal cancers

### 3.1 Gastric cancer

The effects and mechanisms of tonics on gastric cancer *via* OMICs are listed in Table 1.

**TABLE 1:** Applications of OMICs on tonics as adjuvants in gastric cancer.

Compound, herb, and formula	Tonics (in formula)	Study form	OMICs and role	Dose or concentration	Mechanism	Targeted hallmark	Reference
Guiqi Baizhu	Angelicae Sinensis Radix^a^, Astragali Radix^a^, Atractylodis macrocephalae^a^, Paeoniae Radix Alba^a^, and Glycyrrhizae Radix et Rhizoma Praeparata cum Melle^a^	*In vitro*	Genomics and network analysis for identifying the active compounds	570.07 nmol/L	↓HER-2 and PD-L1	Sustaining proliferative signaling pathways	Li et al. (2021a)
N-butylidenephthalide (BP)	Angelicae Sinensis Radix^a^	*In vitro* and *in vivo*	Transcriptomics combined with qPCR, WB, and siRNA transfection for studying effects and mechanisms	*In vitro*: 50 μg/ml AGS or BP 75 μg/ml; and *in vivo*: 300, 500, and 700 mg/kg	↑REDD1; ↓ mTOR signaling	Sustaining proliferative signaling pathways	Liao et al. (2018)
Ginsenoside F2	Ginseng Radix et Rhizoma^a^	*In vitro*	Proteomics for screening the signaling pathways	20 μM	↑p53 and Bcl-xl/Beclin-1	Resisting cell death	Mao et al. (2016)
Dendrobium extract (DOE)	Dendrobium officinale Kimura et Migo (Tiepishihu)^a^	*In vitro*	Metabolomics with qPCR for screening the metabolomic and signaling pathways	DOE (polysaccharides 45%)	↓VEGF, ↓SPHK1, and ↓S1PR1 mRNA by metabolite sphingosine-1-phosphate (S1P)	Inducing angiogenesis	Zhao et al. (2017)
18β-Glycyrrhetinic acid (GRA)	Glycyrrhizae Radix et Rhizoma^a^	*In vitro*, *in vivo*, and human GC tissue collection	Genomics with qRT-PCR for screening the methylation genes and targeted gene	50–200 μM for *in vitro* and 0.05% GRA for *in vivo*	↑ ATP4a activation and ↓ DNMT1	Genome instability mutation	Cao et al. (2020)
Paeonol	*Paeonia lactiflora* Pall.^a^	*In vitro* and *in vivo*	Genomics with qPCR, CCK-8, and TUNEL for studying the synergistic mechanisms	*In vitro*: 60 mg/L and *in vivo*: 30 and 50 mg/kg/d i.p	↓LINC00665 and MAPK1 and ↑miR-665	Enhancing therapeutic effects and/or decreasing side effects *via* drug interactions	Li et al. (2022b)
Jianpi Yangzheng Xiaozheng recipe	Astragali Radix^a^, Codonopsis Radix^a^, Atractylodis Macrocephalae Rhizoma^a^, Dioscoreae Rhizoma (whole herbs of *Hedyotis diffusa* Willd, Baihuasheshecao), Angelicae Sinensis Radix^a^, Paeoniae Radix Alba^a^, and Glycyrrhizae Radix et Rhizoma^a^	*In vitro*	Metabolimics for screening the metabolomic pathways	37.15 and 74.30 g/kg	↑ arachidonic acid and α-linolenic acid and ↑ α-linolenic acid and linoleic acid metabolic pathway	Enhancing body resistance to carcinogenesis	Xu et al. (2021)
Yiqi Fusheng recipe	Atractylodis Macrocephalae Rhizoma^a^, Astragali Radix^a^, Myristicae Semen, Codonopsis Radix^a^, Poria, and Akebiae Fructus	*In vitro*	Metabolomics for screening the metabolomic pathways	1 g/ml	↓ (3-hydroxybutyric acid, methionine, valine, and glutamine), ↑ (low density lipoprotein/LDL/ very low density lipoprotein VLDL, glutamic acid, triglycerides, unsaturated fatty acids, and choline)	Deregulating cellular energetics	He et al. (2016)
Yiwei decoction	*Astragalus membranaceus* (Fisch.) Bge. var. mongholicus (Bge.) Hsiao (Huangqi)^a^, *Ophiopogon japonicus* (L. f) Ker-Gawl. (Maidong)^a^, *Tetrastigma hemsleyanum* Diels et Gilg (Sanyeqing), *Pinellia ternate* (Thunb.) Breit. (Banxia), *Taraxacum mongolicum* Hand.-Mazz. (Pugongying), *Paeonia lactiflora* Pall. (Shaoyao)^a^, *Actinidia chinensis* Planch. (Tengligen), *Coix lacryma-jobi* L. var. ma-yuen (Roman.) Stapf (Yiyiren), and *Rabdosia amethystoides* (Benth.) Hara. (Xiangchacai)	*In vivo*	Metabolomics and bioinformatics for studying the energetic signaling pathways	1.09 g/ml	Intervened gastric precancerous lesions *via* regulating 13 metabolites that involved in the biosynthesis of unsaturated fatty acids, biosynthesis of valine, leucine and isoleucine, sphingolipid metabolism, arachidonic acid metabolism, and steroid hormone synthesis	Deregulating cellular energetics	Dong et al. (2020)
Dendrobium extract (DOE)	*Dendrobium officinale* Kimura et Migo (Tiepishihu)	*In vivo*	Metabolomics for studying the energetic signaling pathways	0.06–0.24 g/kg	DOE can block the progression of gastric precancerous lesions, its mechanism may be related to porphyrin metabolism, tryptophan metabolism, folic acid and pterin biosynthesis, galactose metabolism, and arachidonic acid metabolism	Deregulating cellular energetics	Wang et al. (2018)

^a^ Tonics.

↑: induction, upregulation, or activation; ↓: reduction, downregulation, or inactivation.

#### 3.1.1 Proteomics

Ginsenosides are the major bioactive constituents in ginseng (roots and rhizomes *Panax ginseng* C. A. Mey.), a famous Qi-tonifying CM. Among these, ginsenoside F2 possesses anticancer effects in the human gastric carcinoma cell line SGC7901 (Mao et al., 2016). An iTRAQ-based proteomic analysis in combination with western blotting (WB) revealed that ginsenoside F2 induced autophagic cell death in the human gastric carcinoma cell line SGC7901 *via* an increase in Atg5, Atg7, Atg10, and PUMA, the ribosomal protein-p53 signaling pathway, and Beclin-1, UVRAG, and AMBRA-1, important molecules in the Bcl-xl/Beclin-1 pathway (Mao et al., 2016).

#### 3.1.2 Transcriptomics

Angelicae Sinensis Radix (roots of *Angelica sinensis* (Oliv.) Diels, Danggui) is a blood-tonifying CM (Xu et al., 2018). It was reported that Angelicae Sinensis Radix can treat patients with gastric cancer. The transcriptomic results showed that n-butylidenephthalide, the active compound in Angelicae Sinensis Radix, induced REDD1 (regulated in development and DNA damage responses 1) and consequently inhibited its downstream factor mammalian target of rapamycin (mTOR) in gastric cancer (Liao et al., 2018).

#### 3.1.3 Genomics

The effects and the substantial basis of Guiqi Baizhu prescription, a complex formula, including Angelicae Sinensis Radix, Astragali radix (roots of *Astragalus membranaceus* (Fisch.) Bge. var. mongholicus (Bge.) Hsiao and *Astragalus membranaceus* (Fisch.) Bge., Huangqi), Atractylodis macrocephalae Rhizoma (rhizomes of *Atractylodes macrocephala* Koidz., Baizhu), Paeoniae Radix Alba (roots of *Paeonia lactiflora* Pall., Baishao), Pericarpium Citri Reticulatae (peels of *Citrus reticulata* Blanco, Chenpi), Rhei Radix et Rhizoma (roots and rhizomes of *Rheum palmatum* L., *Rheum tanguticum* Maxim. ex Balf. and *Rheum officinale* Baill, Dahuang), and Glycyrrhizae Radix et Rhizoma Praeparata cum Melle (processed roots and rhizomes of *Glycyrrhiza uralensis* Fisch., *Glycyrrhiza inflata* Bat., or *Glycyrrhiza glabra* L., Zhigancao), are remained to be explored. The genomic assay combined with network pharmacology showed that quercetin, daidzein, and isorhamnetin had potential antiproliferative effects on HER-2 and PD-L1 in human gastric cancer (GC) MKN-45 cells. Quercetin, daidzein, and isorhamnetin are the components in Astragoli Radix, indicating that Astragali Radix instead of Angelicae Sinensis Radix played the main role in the proliferation of GC (Li et al., 2021a), although Astragali Radix may have a synergistic effect in the formula (Liao et al., 2018).

Another role of genomics is in the shortage of chemotherapy—drug resistance. How to reverse drug resistance *via* OMICs has drawn attention from the medical community. Paeoniae Radix Alba is one of the blood tonics used for nourishing the blood and regulating menstruation, astringing Yin and checking sweeting, emolliating the liver, relieving pain, and depressing the liver Yang. Although Paeoniae Radix Alba reversed the drug resistance of GC cells, the mechanism was unknown until paeonol (the active compound in Paeoniae Radix Alba) was reported using genomics—data showed that paeonol inhibited the malignancy of apatinib-resistant GC cells through the LINC00665/miR-665/MAPK1 axis (Li et al., 2022b).

#### 3.1.4 Metabolomics

*Dendrobium officinale* Kimura et Migo (Tiepishihu) is one of the sources of Dendrobii Caulis (Shihu). To investigate the substantial basis of Dendrobii Caulis on gastric cancer, blood metabolites were analyzed to screen the active compounds using UPLC-Q-TOF-MS. The metabolomics results showed that among five candidate metabolites, phingosine-1-phosphate (S1P) inhibited GC angiogenesis by inhibiting VEGF, SPHK1, and S1PR1 mRNA in rats (Zhao et al., 2017). Another urine metabolomics study showed that Dendrobii Caulis aqueous extracts inhibited the progression of gastric precancerous lesions. The mechanism is related to porphyrin metabolism, tryptophan metabolism, folic acid and pterin biosyntheses, and galactose and arachidonic acid metabolisms (Wang et al., 2018).

Yiwei decoction is a tonic formula that includes *Astragalus membranaceus* (Fisch.) Bge. var. mongholicus (Bge.) Hsiao (Huangqi), *Ophiopogon japonicus* (L. f) Ker-Gawl. (Maidong), *Tetrastigma hemsleyanum* Diels et Gilg (Sanyeqing), *Pinellia ternate* (Thunb.) Breit. (Banxia), *Taraxacum mongolicum* Hand.-Mazz. (Pugongying), *Paeonia lactiflora* Pall. (Shaoyao), *Actinidia chinensis* Planch. (Tengligen), *Coix lacryma-jobi* L. var. ma-yuen (Roman.) Stapf (Yiyiren), and *Rabdosia amethystoides* (Benth.) Hara. (Xiangchacai). The serum metabolomics results showed that Yiwei decoction intervened in gastric precancerous lesions by regulating 13 metabolites involved in the biosynthesis of unsaturated fatty acids, biosynthesis of valine, leucine, and isoleucine, sphingolipid metabolism, arachidonic acid metabolism, and steroid hormone synthesis (Dong et al., 2020). The Yiqi Fusheng recipe includes Atractylodis Macrocephalae Rhizoma, Astragali Radix, Myristicae Semen (seeds of *Myristica fragrans* Houtt., Roudoukou), Codonopsis Radix (roots of *Codonopsis pilosula* (Franch.) Nannf., *Codonopsis pilosula* Nannf. var. modesta (Nannf.) L. T. Shen, and *Codonopsis tangshen* Oliv., Dangshen), Poria (sclerotium of *Poria cocos* (Schw.) Wolf (Fuling), and Akebiae Fructus (immature fruits of *Abebia quinate* (Thunb.) Decne., *Akebia trifoliata* (Thunb.) Koidz., *Akebia trifoliata* (Thunb.) Koidz. var. australis (Diels) Rehd., Yuzhizi). The metabolomics results showed that the Yiqi Fusheng recipe can be used to treat spleen-Qi deficient mice with gastric cancer by targeting energy metabolism reprogramming. The mechanisms may lie in lowering the content of 3-hydroxybutyric acid, methionine, valine, and glutamine, and increasing low density lipoprotein (LDL)/very low density lipoprotein (VLDL), glutamic acid, triglycerides, unsaturated fatty acids, and choline (He et al., 2016).

The Jianpi Yangzheng Xiaozheng recipe comprises Astragali Radix, Atractylodis Macrocephalae Rhizoma, Codonopsis Radix, Poria, Dioscoreae Rhizoma (rhizomes of *Dioscorea opposita* Thunb., Shanyao), Coicis Semen (seeds of *Coix lacryma-jobi* L. var. *ma-yuen* (Roman.) Stapf, Yiyiren), Citri Reticulatae Pericarpium, Aucklandiae Radix (roots of *Aucklandia lappa* Decne., Muxiang), Angelicae Sinensis Radix, Paeoniae Radix Alba, Smilacis Chinae Rhizoma (rhizomes of *Smilax china* L., Baqia), Salviae Chinensis Herba (whole herbs of *Salvia chinensis* Benth., Shijianchuan), and Glycyrrhizae Radix et Rhizoma Praeparata Cum Melle. The Jianpi Yangzheng Xiaozheng recipe is reported to enhance body resistance to GC. The serum metabolomics results showed that this effect was associated with an increase in the serum levels of α-linolenic acid, linoleic acid (LA), and arachidonic acid (AA) (Xu et al., 2021). LA can be metabolized to AA. LA/AA plays an important role in enhancing body resistance, i.e., the inflammatory response and immune function (e.g., natural killer cell activity). Although certain arguments show the relationship between LA/AA and breast cancer (Gago-Dominguez et al., 2003; Murff et al., 2011), dietary intake of LA/AA was reported to decrease the risk of colorectal cancer (Kuriki et al., 2006) and liver cancer (Bao et al., 2017). This indicates that the Jianpi Yangzheng Xiaozheng recipe may enhance immunofunction through LA/AA metabolism.

### 3.2 Colorectal cancer

OMICs for the adjuvant effects and mechanisms of tonics on colorectal cancer (CRC) are listed in Table 2.

**TABLE 2:** Tonics targeting hallmarks as adjuvants in gastric, liver, and colorectal cancers via OMICs.

Compound, herb, and formula	Tonics (in formula)	Cancer	Study form	OMICs and role	Dose or concentration	Mechanism	Targeted hallmark	Reference
Ginsenoside-Rp1	Ginseng Radix et Rhizoma^a^	Colorectal	*In vitro*	Proteomics with proliferation assay and propidium iodine staining for screening the signaling pathway	60 mM	↑ Apo-A1	Sustaining proliferative signaling pathways	Kim et al. (2014)
Gegen Qinlian decoction (GQD)	Puerariae Lobatae Radix, Scutellariae Radix, Coptidis Rhizoma, and Glycyrrhizae Radix et Rhizoma^a^	Colon	*In vitro*	Transcriptomics and network pharmacology for screening the drug compatibility and the signaling pathway	GLY-PUE combination (GLY, 60 and 70 μM)	↑GSK3B and ↓CTNNB1	Sustaining proliferative signaling pathways	Li et al. (2021b)
Jujube polysaccharides	*Zizyphus jujuba* cv. Muzao^a^	Colorectal	*In vivo*	Metabolomics and transcriptomics for screening the effects on metabolisms and the gut microbiota	200 and 1,000 mg/kg	↑ short-chain fatty acids (SCFAs) and *Bifidobacterium*, *Bacteroides*, and *Lactobacillus*	Inflammation-mediated carcinogenesis	Ji et al. (2019)
American ginseng	Panacis Quinquefolii Radix^a^	Colon	*In vivo*	Metabolomics for screening the dysregulated metabolism pathways	10 and 20 mg/kg/d	↓ (IL-1α, IL-1β, IL-6, G-CSF, and GM-CSF); ↑ (arachidonic acid, linolelaidic acid, glutamate, docosahexaenoate, tryptophan, and fructose)	Inflammation-mediated carcinogenesis	Xie et al. (2015)
Jujube polysaccharides	Jujubae Fructus^a^	Colorectal	*In vivo*	Transcriptomics for screening the effects on the gut microbiota	1,000 mg/kg/d	↑ short-chain fatty acids (SCFAs) and ↓ *Firmicutes/Bacteroidetes*	Inflammation-mediated carcinogenesis	Ji et al. (2020)
American ginseng	Panacis Quinquefolii Radix^a^	Colon	*In vitro* and *in vivo*	Metabolomics and transcriptomics for studies on restoring the metabolomic and microbiota profiles	15 and 30 mg/kg/d	↓ (1L-1a, 1L-1B, 1L-6, G-CSF, GM-GSF), ↓ malic acid and 2-hydroxybutanoic acid, and ↓ *Bacteroidetes* and *Verrucomicrobia*	Inflammation-mediated carcinogenesis	Wang et al. (2016a)
*Glycyrrhiza* polysaccharide (GCP)	*Glycyrrhiza Uralensis* Fisch.^a^	Colon	*In vivo*	Transcriptomics with HE staining for screening the effects on the gut microbiota	500 mg/kg	↑ (*Enterorhabdus*, Odoribacter, *Ruminococcaceae*_UCG_014, *Ruminococcaceae*_UCG_010, *Enterococcus*, *Ruminiclostridium*_5), and ↓ (*Parasutterella*, *Clostridium*_sensu_stricto_1, *Blautia*)	Inflammation-mediated carcinogenesis	Zhang et al. (2018)
Isoliquritigenin (ILTG)	*Glycyrrhiza glabra* L.^a^	Colon	*In vitro*	Epigenomics with cytotoxicity assay and an ethidium bromide displacement assay for screening the methylation genes	11.1 μg/ml	↓DNA methylation	Genomic instability and mutation	Zorko et al. (2010)
*Astragalus* membranaceus extract	*Astragalus membranaceus* (Fischer) Bge. var. mongolicus (Bge.) Hsiao (AM)^a^	Colorectal	*In vivo*	Transcriptomics for screening the mechanisms	500 mg/kg/d	Regulating epigenetic-related genes including KMT2D, BRD2, CREBBP, and ARID1A	Genome instability mutation	Tseng et al. (2016)
Compound K	Ginseng Radix et Rhizoma^a^	Colon	*In vitro*	Genomics for screening the signaling pathways	20 ± 1.0 μg/ml	↓histone deacetylase (HDAC) activity, mRNA, and protein expression. ↑RUNX3 and p21	Genome instability mutation	Kang et al. (2013)
Daikenchuto (DKT)	Ginseng Radix et Rhizoma^a^	Colon	Clinical study (after laparoscopic colectomy)	Metabolomics and transcriptomics for screening the effects on metabolomic pathways and gut microbiota	5g, t.i.d	↓arachidonic acid cascade and ↓*Serratia* and *Bilophila*	Enhancing body resistance by reduction gastrointestinal symptoms	Hanada et al. (2021)
Quxie capsules	Ginseng Radix et Rhizoma^a^, Zingiberis Rhizoma, Aquilariae Lignum Resinatum, Crotonis Fructus, Gleditsiae Spina	Colorectal	Clinical study (after chemotherapy, radiotherapy, targeted therapy, and immunotherapy)	Metabolomics and transcriptomics for screening the effects on metabolomic pathways and gut microbiota	0.05 g/kg, b.i.d	Improving beneficial bacteria in the intestinal tract and reducing the distribution ratio of harmful bacteria *via* modulating nicotinic acid and nicotinamide, anthocyanin and tryptophan metabolism pathway	Enhancing body resistance to carcinogenesis	Sun et al. (2021a)
*Astragalus* membranaceus-Curcuma wenyujin (AC)	*Astragalus* membranaceus^a^	Colorectal	*In vivo*	Metabolomics for screening the drug compatibility and the signaling pathway and the energetic signaling pathways	AC at the ratio of 2:1	↓(valine, leucine, and isoleucine biosynthesis, aminoacyl-tRNA biosynthesis, caffeine metabolism pathway, and retinol metabolism pathways)	Activating invasion and metastasis and deregulating cellular energetics	Sun et al. (2021b)
Polysaccharides and ginsenosides	American Ginseng (*Panax quinquefolius* L.)^a^	Gastrointestinal	*In vitro* and *in vivo*	Metabolomics and transcriptomics for studying synergistic mechanisms	1,500 mg/kg/d) + ginsenoside (150 mg/kg/d, AGP_AGG	↓ CTX-induced intestinal immune disorders and gut barrier dysfunctions	Enhancing body resistance to carcinogenesis	Zhou et al. (2021a)

^a^ Tonics.

↑: induction, upregulation, or activation; ↓: reduction, downregulation, or inactivation.

#### 3.2.1 Proteomics

Proteomic data showed that 20S-ginsenoside Rg3, an active compound in ginseng, induced colon cancer apoptosis by downregulating the Rho GDP dissociation inhibitor (RhoGDI), together with upregulating tropomyosin 1, annexin 5, and glutathione s-transferase p1 (GSTP1) (Lee et al., 2009).

#### 3.2.2 Transcriptomics

There is an interesting concept, namely, the Chinese herb pair (Yao Dui). In this case, two herbs are commonly included at an appropriate ratio in some formulas for enhancing effects and/or decreasing toxicity. Gegen Qinlian decoction (GQD) is an ancient formula from the Han dynasty. Since it consists of four herbs, Puerariae Lobatae Radix (roots of *Pueraria lobata* (Willd.) Ohwi, Gegen), Scutellariae Radix (roots of *Scutellaria baicalensis* Georgi, Huang Qin), Coptidis Rhizoma (rhizomes of *Coptis chinensis* Franch., *Coptis deltoidea* C. Y. Cheng et Hsiao and *Coptis teeta* Wall., Huanglian), and Glycyrrhizae Radix et Rhizoma (roots and rhizomes of *Glycyrrhiza uralensis* Fisch., *Glycyrrhiza inflata* Bat., or *Glycyrrhiza glabra* L., Gancao), the dominant herbs have not yet been clarified. The transcriptomics data by Li et al. (2021b) showed that two active compounds, puerarin (PUE) and glycyrrhetinic acid (GLY) without other active compound pairs, influenced the Wnt signaling pathway by upregulating GSK3B and downregulating CTNNB1 synergistically in colon SW480 cells. As PUE and GLY are the main components of Puerariae Lobatae Radix and Glycyrrhizae Radix et Rhizoma, respectively, the results confirmed the pharmacological role of the herb pair, Puerariae Lobatae Radix and Glycyrrhizae Radix et Rhizoma, in GQD.

#### 3.2.3 Genomics

Accumulating evidence suggests that aberrant DNA methylation and gene silencing of tumor suppressors are pervasive in GI cancers (Cao et al., 2020). ATP4a is an important tumor suppressor gene, encoding H^+^, K^+^-ATPase, and there is an inverse correlation between methylation and expression in ATP4a. Genomics evidence showed that isoliquritigenin (ILTG), an active compound in *Glycyrrhiza glabra* L., exhibited a demethylating activity on HT-29 colon cancer by increasing ATP4a (Zorko et al., 2010).

#### 3.2.4 Metabolomics

*Astragalus membranaceus* and *Curcuma wenyujin* (AC) are classic Chinese herb pairs for colon cancer metastasis. Metabolomics data indicate that AC inhibits liver and spleen metastases of colon cancer by disturbing energetic dysfunction including valine, leucine, and isoleucine biosyntheses, aminoacyl-tRNA biosynthesis, caffeine metabolism pathway, and retinol metabolism pathways (Sun et al., 2021b).

The metabolomic studies showed that Panacis Quinquefolii Radix (roots of *Panax quinquefolium* L., American ginseng or Xiyangshen) attenuated colitis-associated colon carcinogenesis in mice *via* a decrease in the inflammatory cytokines IL-1α, IL-1β, IL-6, G-CSF, and GM-CSF. Panacis Quinquefolii Radix also decreased the impaired metabolism of arachidonic acid, linolelaidic acid, glutamate, docosahexaenoate, tryptophan, and fructose, all of which are associated with inflammation and oxidation (Xie et al., 2015).

Quxie capsules (QXC) are the adjuvant drugs for CRC to reduce intestinal complication, which comprise Ginseng Radix et Rhizoma, Zingiberis Rhizoma (rhizomes of *Zingiber officinale* Rose., Ganjiang), Aquilariae Lignum Resinatum (resinatum of *Aquilaria sinensis* (Lour.) Gilg, Chenxiang), Crotonis Fructus (fruits of *Croton tiglium* L., Badou), and Gleditsiae Spina (fruits of *Gleditsia sinensis* Lam., Dazaojiao). The serum metabolomics data showed that QXC improved beneficial bacteria in the intestinal tract and reduced the distribution ratio of harmful bacteria by modulating the nicotinic acid and nicotinamide, anthocyanin, and tryptophan metabolism pathways in patients with CRC (Sun et al., 2021a).

#### 3.2.5 Multi-OMICs

Jujubae Fructus (Dazao) is the fruit of *Zizyphus jujuba* Mill. for tonifying Qi in CMs. The transcriptomic and metabolomic profiles showed that its polysaccharide consumption prevented mouse CRC and decreased colon mortality, reduced proinflammatory cytokines, increased the concentration of total short-chain fatty acids (SCFAs) and gut microbiota *Bifidobacterium*, *Bacteroides*, and *Lactobacillus*, and decreased gut microbiota *Firmicutes/Bacteroidetes* in mouse feces, indicating that Jujubae Fructus polysaccharides prevented inflammation-mediated carcinogenesis by restoring the balance of the gut microbiota in CRC (Ji et al., 2019; Ji et al., 2020).

Cyclophosphamide (CTX) is a widely used chemotherapy drug. However, it may result in complicated adverse effects including vomiting, diarrhea, and abdominal pain, related to the disruption of the mucosal barrier, bacterial translocation, and changes in microbial composition. Polysaccharides and ginsenosides are the two classes of active compounds in American ginseng. Through metabolomic and transcriptomic analyses, these polysaccharides and ginsenosides were found to exert synergistic effects to ameliorate CTX-induced intestinal immune disorders and gut barrier dysfunctions (Zhou et al., 2021a).

### 3.3 Liver cancer

OMICs for the adjuvant effects and mechanisms of tonics on liver cancer are listed in Table 3.

**TABLE 3:** Applications of OMICs on tonics as adjuvants in liver cancer.

Compound, herb, and formula	Tonics (in formula)	Study form	OMICs and role	Dose or concentration	Mechanism	Targeted hallmark	Reference
Xiaoai Jiedu recipe	Hedyotis diffusa, Scutellaria barbata Herba (whole herbs of *Scutellaria barbata* D. Don, Banzhilian), Dioscoreae Rhizoma, Curcumae Rhizoma, Cremastrae Pseudobulbus Pleiones Pseudobulbus (*Iphigenia indica* Kunth, Shancigu), Pseudostellariae Radix (*Pseudostellaria heterophylla* (Miq.) Pax ex Pax et Hoffm., Taizishen)^a^, Ophiopogonis Radix (*Ophiopogon japonicus* (L. f) Ker-Gawl., Maidong)^a^	*In vivo*	Proteomics for screening the signaling pathway	1.93 and 3.86 g/ml	Regulating glutathione metabolism, PPAR, toll-like receptors, HIF-1, NF-κB, mTOR, and p53 signaling pathway	Sustaining proliferative signaling pathways	Rao et al. (2018)
Daidzein	Astragali Radix^a^	*In vitro*	Transcriptomics for screening the signaling pathways	NA	↑HMOX1, ↓ (MT1G, MT1X, and MT1F), and ↑ferroptosis	Resisting cell death	Liu et al. (2021b)
*Astragali Radix protein* extract	*Astragalus membranaceus* (Fisch.)^a^	*In vitro*	Transcriptomics with flow cytometry, WB, qPCR, and Hoechst/propidium iodide for screening the signaling pathways	10, 50, and 100 μg/ml	↑ necrosis *via* regulating homophilic cell adhesion *via* plasma membrane adhesion molecules, P53 downstream pathway, response to endoplasmic reticulum stress, and steroid metabolic process	Resisting cell death	Wang et al. (2020b)
Cordycep sinensis	*Cordyceps sinensis* (BerK.) Sacc.^a^	*In vivo*	Proteomics with histopathological analysis for screening the mechanisms	NA	Regulating oxidative stress and detoxification including catalase, DHE3 (glutamate dehydrogenase 1), PRDX1 (peroxiredoxin-1), GSTP (glutathione S-transferase P), and GSTM1 and GSTM2	Genome instability mutation	Wang et al. (2016b)
Jiawei Xiaoyao pulvis	Bupleuri radix (roots of *Bupleurum chinense* DC. or *Bupleurum scorzonerifolium* Willd., Chaihu), Angelicae Sinensis Radix (Danggui) ^a^, Paeoniae radix rubra (Chishao), Paeoniae radix alba (Baishao) ^a^, Codonopsis radix (Dangshen) ^a^, Atractylodis macrocephalae rhizoma (Baizhu) ^a^, Glycyrrhizae Radix et Rhizoma (Gancao) ^a^, Salviae miltiorrhizae radix et rhizoma (roots of Salvia miltiorrhiza Bge., Danshen), Hordei Fructus Germinatus (germinatus of *Hordeum vulgare* L., Maiya), Setariae Fructus Germinatus (germinatus of *Setaria italica* (L.) Beauv., Guya), Corydalis Rhizoma (rhizomes of *Corydalis yanhusuo* W. T. Wan, Yanhusuo), Moutan Cortex (cortex of *Paeonia suffruticosa* Andr., Mudanpi), Poria (Fuling), Coicis Semen (Yiyiren), Gardeniae Fructus (fruits of *Gardenia jasminoides* Ellis, Zhizi), Aurantii Fructus Immaturus (immature fruits of *Citrus aurantium* L. or *Citrus sinensis* Osbeck, Zhishi), and Citri Reticulatae Pericarpium (Chenpi)	*In vivo*	Proteomics with network pharmacology and HE and ELISA for screening the signaling pathways	3.55 g/ml	↓liver carcinogenesis-induced depression	Enhancing body resistance to carcinogenesis	Wen et al. (2022)
Sijunzi decoction	Ginseng Radix et Rhizoma ^a^, Atractylodis Macrocephalae Rhizoma ^a^, Poria and Glycyrrhizae Radix et Rhizoma ^a^	Clinical study (the primary therapy was not given)	Metabolomics for screening the effects on metabolomic pathways	NA	Regulating the metabolisms of amino acids, arachidonic acid, fatty acids, and glutathione	Enhancing body resistance to carcinogenesis	Wang et al. (2020a)
Jianpi Jiedu recipe	Codonopsis Radix ^a^, Poria, Atractylodis Macrocephalae Rhizoma ^a^, Glycyrrhizae Radix et Rhizoma ^a^, Bupleuri Radix, Scutellariae Barbatae herba, Solanum nigrum (whole herbs of *Solanum nigrum* L., Longkui), Portulacae Herba (Aboveground parts of *Portulaca oleracea* L.), and Curcumae Rhizoma	*In vivo*	Metabolomics for studying the energetic signaling pathways	75 g/kg	Regulating valine, leucine, and isoleucine biosytheses, ascorbate and aldarate metabolisms, methane metabolism, glyoxylate and dicarboxylate metabolisms, glycine, serine, and threonine metabolisms, and nicotinate and nicotinamide metabolisms	Deregulating cellular energetics	Liu et al. (2020)
Xiaoai Jiedu recipe	Hedyotis diffusa, Scutellaria barbata Herba, Dioscoreae Rhizoma, Curcumae Rhizoma, Cremastrae Pseudobulbus Pleiones Pseudobulbus, Pseudostellariae Radix^a^, and Ophiopogonis Radix^a^	*In vivo*	Proteomics for studying the energetic signaling pathways	1.93, 3.86 g/ml	Regulating steroid hormones biosynthesis (Cyp2c11, Hsd3b5, Cyp2e1, *etc.*), fatty acid degradation (Acox1, Acsl1, Adh6, *etc.*), and PPAR signaling pathways (Gk, Slc27a5, Lpl, *etc.*)	Deregulating cellular energetics	Xu et al. (2020)
Panax Ginseng	*Panax ginseng* C. A. Mey.^a^	*In vivo*	Metabolomics and transcriptomics for screening the effects on metabolomic pathways and gut microbiota	1.17 g/kg/d	Six major metabolic pathways including bile acid biosynthesis, unsaturated fatty acid biosynthesis, tryptophan metabolism, arachidonic acid metabolism, pyrimidine metabolism, and vitamin B6 metabolism. 23 species of bacteria with significant differences of synergistic action of ginsenosides and polysaccharides	Deregulating cellular energetics	Hou et al. (2022)
Albiflorin	*Paeonia lactiflora* Pall.^a^	*In vitro*	Transcriptomics with CCK-8, and transwell assay for screening the mechanisms	200 μmol/L	Regulate BDKRB1, TUBA8, ADRB1, and GRM1	Activating invasion and metastasis and deregulating cellular energetics	Zhou et al. (2021b)

^a^ Tonics.

↑: induction, upregulation, or activation; ↓: reduction, downregulation, or inactivation.

#### 3.3.1 Proteomics

Concerning complications, patients with liver cancer may suffer from depression, which is also an inducing factor in hepatocellular carcinogenesis. The Jiawei Xiaoyao pulvis is a formula and was reported as an adjuvant to relieve liver carcinogenesis-induced depression. The proteomic results showed that Jiawei Xiaoyao pulvis reversed depression-like behaviors by regulating GSTM1, PDK1, and HSP90AB1 (Wen et al., 2022) (Table 3).

#### 3.3.2 Transcriptomics

*Astragalus* protein is the active compound from the tonics *Astragalus membranaceus* (Fisch.). Transcriptomic data in combination with qRT-PCR and WB showed that *Astragalus* protein induced programmed necrosis of liver cancer HepG2 cells *via* the p53 signaling pathway (Wang et al., 2020b). Transcriptomics and network pharmacology studies have shown that daidzein (an active compound in Astragali Radix) induces ferroptosis by downregulating MT1G in liver cancer (Liu et al., 2021b).

#### 3.3.3 Metabolomics

According to Chinese medicine philosophy, one of the typical syndromes of liver cancer is spleen deficiency, with which patients may suffer from cancer pain, ascites, fatigue, *etc.* (Xu et al., 2018). Sijunzi decoction (Ginseng Radix et Rhizoma, Atractylodis Macrocephalae Rhizoma, Poria, and Glycyrrhizae Radix et Rhizoma) is a tonifying formula for treating spleen deficiency. However, because of the complex mixture of multiple components and multiple targets, how to profile spleen deficiency and how to interpret Chinese herbs treating such a syndrome pharmacologically are challenges. Based on blood plasma metabolomics using UPLC-HDMS, Wang et al. found that Sijunzi decoction treated spleen deficiency with metabolic dysfunctions in liver cancer *via* regulating the metabolisms of amino acids, arachidonic acid, fatty acids, and glutathione (Wang et al., 2020a).

#### 3.3.4 Multi-OMICs

Using metabolomics for fecal metabolites and transcriptomics for gut microbiota, Panax Ginseng was found to regulate bile acid biosynthesis, unsaturated fatty acid biosynthesis, tryptophan metabolism, arachidonic acid metabolism, pyrimidine metabolism, and vitamin B6 metabolism. Furthermore, 25 species of bacteria with significant differences in effective parts in liver cancer and 23 species of bacteria with significant differences in synergistic action of ginsenosides and polysaccharides indicated that Qi deficiency liver cancer was associated with bile acid biosynthesis, unsaturated fatty acid biosynthesis, tryptophan metabolism, arachidonic acid metabolism, pyrimidine metabolism, vitamin B6 metabolism, and certain gut microbiota (Hou et al., 2022).

## 4 Discussion

**FIGURE 2:**
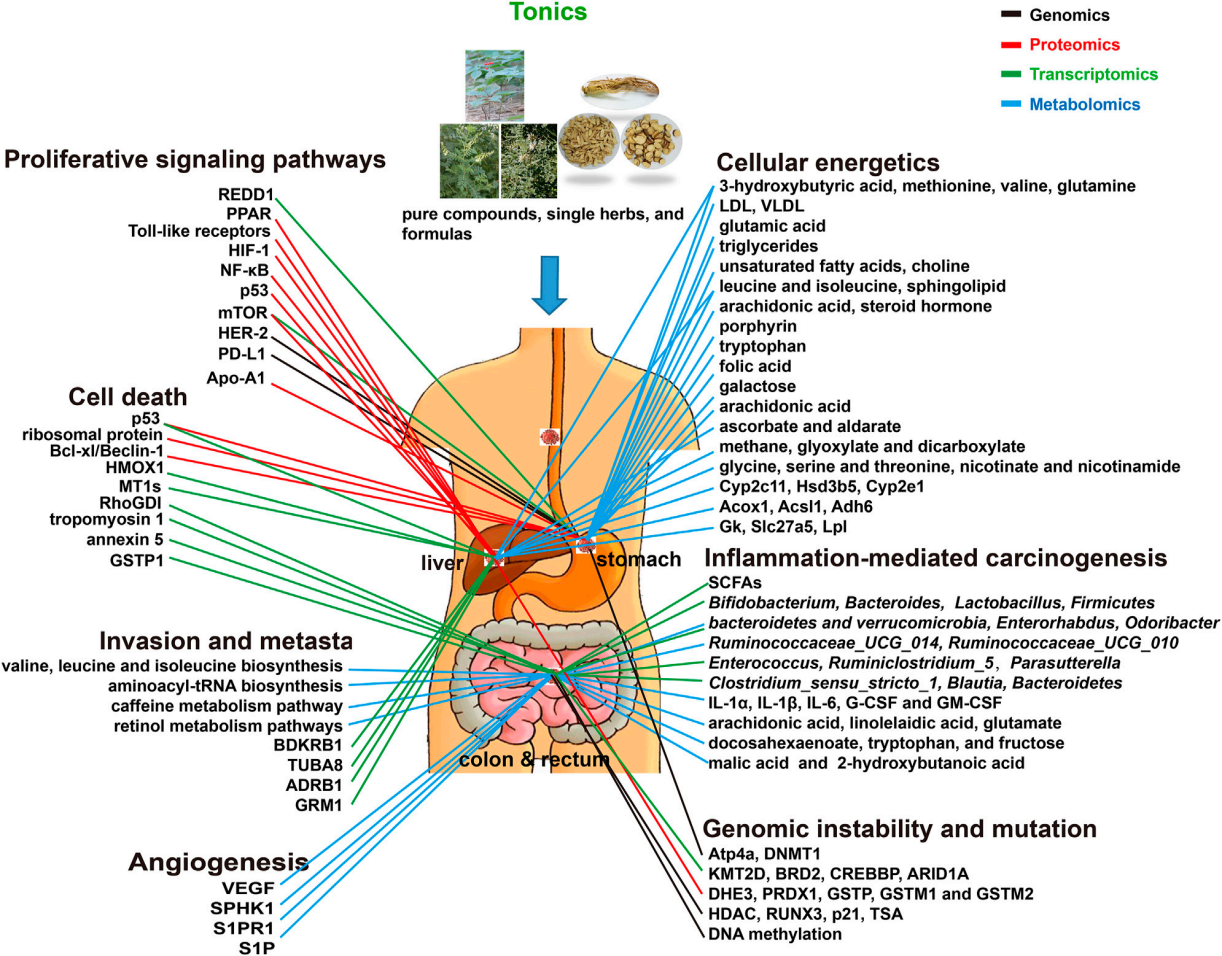
Tonics targeting hallmarks as adjuvants in gastric, liver, and colorectal cancers *via* OMICs.

Due to the heterogeneity of cancer, different molecular targets achieve different effects, and even cancers of one organ require different treatment strategies. This leads to the challenges for anti-GI cancers, especially in terms of understanding their underlying mechanisms. Thus, Hanahan introduced a new concept, hallmark, to distinguish the different mechanisms and potential targets for anticancer (Hanahan and Weinberg, 2011). Tonics are effectively complementary and alternative medicines to conventional treatments with less toxicity. OMICs are novel technologies with a small sample size, and large-scale and high-throughput screening. These properties make it possible to apply OMICs for exploring the pharmacological mechanisms of tonics, especially targeting cancer hallmarks. In this study, the data showed that the targets of tonics included sustaining proliferative signaling, resisting cell death, activating invasion and metastasis, inducing angiogenesis, deregulating cellular energetics, inflammation-mediated carcinogenesis, and genomic instability and mutation (Table 1, Table 2, and Table 3; Figure 2).

However, there are still some challenges to limit the applications of OMICs. To further explore the role of OMICs in tonics on GI cancer, we focused on the active compounds, mechanisms, and compatibility of tonics, combined with basic experiments and novel technologies, and emphasized minimal injury methodologies.

First, tonics in clinical use are herbs, especially in formulas. This means that tonics for anti-GI cancer have multiple components and targets (signaling pathways) and complex mixtures. Thus, OMICs for tonics in anti-GI cancer research should focus on the active compounds, mechanisms, and compatibility of tonics to determine the relationship between the active compounds of tonics and their effects and underlying mechanisms, e.g., using comprehensive two-dimensional liquid chromatography (2DLC), Qiao et al. found 311 compounds from the extract of *Glycyrrhiza uralensis* Fisch. within 40 min, of which the method was superior to high-performance liquid chromatography (HPLC). Then, chemicalomics and metabolomics are matched, where metabolomics facilitates the exploration of the active compounds and analysis of the responding signaling pathways *via* metabolites, while transcriptomics easily unveils the differentially expressed genes *via* RNA sequencing (Gong et al., 2012). Another strategy for exploring the compound–herb–disease relationship is polypharmacokinetics (Poly-PK), a novel technology for OMICs comparison. For example, Xie et al. identified 84, 292 and 532 compounds in extracts of Huang Qin decoction (including *Scutellaria baicalensis* Georgi and *Glycyrrhiza uralensis* Fisch.) and serum metabolites before and after oral administration, respectively. Among these compounds, 485 were changed after oral administration, of which 56 were from the extract of Huang Qin decoction, 292 were metabolites in the PK process, and 166 were metabolites from endogenous components. This methodology may profile a complex network between tonics, drug metabolites, and body metabolism function and provide insights into the holistic effect of a complex of tonics formulas (Xie et al., 2018).

Second, there are often inconsistencies between different results of OMICs, at least partially caused by the small size of the sample and systematic deviation. It is necessary to integrate basic experimental methods such as real-time PCR and WB, to confirm OMICs data and obtain more accurate results. Additionally, only genomics, transcriptomics, epigenomics, metabolomics, and proteomics among OMICs were used in this study, while glycomics and lipomics were not applied. Moreover, with the development of modern technologies, novel methods can be introduced in the research of tonics on GI cancer. For example, hepatocellular carcinoma (HCC) is characterized by high heterogeneity and metastatic potential and leads to poor prognosis. Thus, single-cell transcriptomic and proteomic data may identify mutations in small populations of cells and distinguish metastatic potential cells from HCC. A number of studies show that Chinese medicines can be used for HCC metastasis (Guo et al., 2022), and most of the mechanisms have been unknown, single-cell multi-OMICs may be a useful tool to unveil the metastatic mechanisms (Peng et al., 2018; Sun et al., 2021c; Wang et al., 2022) of tonics including *Astragalus membranaceus* (Fisch.) Bge. var. mongholicus (Bge.) Hsiao, *Panax quinquefolium* L., *Atractylodes macrocephala* Koidz., *Glycyrrhiza uralensis* Fisch., and *Polygonum multiflorum* Thunb (Xu et al., 2018, Yang et al., 2022). These potential findings may offer more individual and precise strategies for patients with GI cancer (Lu et al., 2021).

Third, decreased quality of life may worsen for patients suffering from GI cancers, so it is essential to choose simple, non-invasive, or minimally invasive ways for subjects to reduce injuries. The methods include metabolomic or genomic analysis of a small sample of blood, metabolomic analysis of urine, feces, and saliva, or transcriptomics combined with X-ray, computed tomography (CT), magnetic resonance imaging (MRI) scanning, or small animal *in vivo* imaging technology. OMICs in combination with network pharmacology and bioinformatics are another effective way to obtain ideal results (Liu et al., 2021b).

Of note, GI cancers include two families based on disease sites, upper digestive tract cancers (including esophageal, stomach, pancreatic, liver, gallbladder, and lymphoma involving the mucosa-associated lymphoid tissue, gastrointestinal stromal, and biliary tree) and lower cancers (including colorectal, anal, and gastrointestinal carcinoid); however, to the best of our knowledge, only gastric cancer, liver cancer, and colorectal cancer have been reported using tonics *via* OMICs. The literature of tonics on the other kinds of GI cancers may have not been reported using OMICs, or, unfortunately, it may be filtered *via* inclusive and/or exclusive criteria because of low quality of the studies.

Another limitation of this review is that the primary therapies in most studies were not introduced and discussed although most CMs, including tonics, are add-on therapies. In this review, Ginseng Radix et Rhizoma treated colon cancer patients to reduce gastrointestinal symptoms after laparoscopic colectomy (Hanada et al., 2021). The Quxie capsules enhanced body resistance to colorectal cancer after chemotherapy, radiotherapy, targeted therapy, and immunotherapy (Sun et al., 2021a). Polysaccharides and ginsenosides in American Ginseng had effects on CTX-induced intestinal immune disorders and gut barrier dysfunctions (Zhou et al., 2021a) (Table 2). However, for most *in vitro* and *in vivo* studies in this review, in which tonics were used for exploring mechanisms, few primary therapies are discussed. This indicates that high quality studies and more evidence are necessary for tonics as adjuvants. Furthermore, for a high quality of pharmacological study, detailed information (positive and negative controls, minimal active concentration, the model used, concentration or dose, duration, extract process, *in vitro*/*in vivo*/clinical study, *etc.*) are essential. In this study, 12 out of 34 studies (Tables 1, Table 2, and Table 3) were *in vitro*, although they were screened *via* inclusion and exclusion criteria. This may be the result from that the mechanism studies did not involve in *in vivo* and clinical data. However, high-quality studies should be guaranteed.

## 5 Conclusion

With the data from OMICs, tonics were found to be adjuvants for gastric, liver, and colorectal cancers with mechanisms including for targeting cancer hallmarks (sustaining proliferative signaling pathways, resistance to cell death, activation of invasion and metastasis, inducing angiogenesis, deregulating cellular energetics, inflammation-mediated carcinogenesis, genomic instability, and mutation), enhancing body resistance to carcinogenesis, and enhancing therapeutic effects and/or decreasing side effects *via* drug interactions. However, more investigations and evidence are necessary for tonics being used as adjuvants.
